# Ground motion baseline analysis of the Cheshire UK GeoEnergy Observatory

**DOI:** 10.1038/s41598-021-95191-4

**Published:** 2021-08-12

**Authors:** Alessandro Novellino, Luke Bateson, Colm Jordan

**Affiliations:** 1 British Geological Survey, Environmental Science Centre, Keyworth, Nottingham, NG12 5GG UK; 2 Honorary Research Associate with the British Geological Survey, Environmental Science Centre, Keyworth, Nottingham, NG12 5GG UK

**Keywords:** Environmental sciences, Natural hazards

## Abstract

Subsurface geonergy can induce ground motion and seismicity, however a scarcity of observations usually obscures the mechanisms underpinning such behaviour. Here, we analyse Interferometric Synthetic Aperture Radar (InSAR) data from ERS, ENVISAT and Sentinel-1 satellites for the period 1995–2017 and interpret ground deformation in the area of the planned Cheshire UK GeoEnergy Observatory ahead of facility contruction. Ground motion is dominated by the compaction of tidal flat deposits overlying two paleo-valleys, trending NNW–SSE. The western paleo-valley experienced faster subsidence rates in the period 1995–2007, whereas the eastern paleo-valley subsided faster in the period 2016–2017. The research highlights how baseline assessment can help differentiate natural variation from any anthropogenic effects associated with the growth of new subsurface technologies.

## Introduction

The UK Geoenergy Observatories (UKGEOS) project is a £31 m UK government investment in at-scale research infrastructure to support the development of new, low carbon, subsurface energy supply and storage technologies. The funding is being used to develop two subsurface research sites, known as the Glasgow and Cheshire Observatories and a new laboratory equipped with a suite of core scanning instruments. Together these will deliver free, open and accessible data on the response of the subsurface to energy storage. The British Geological Survey (BGS) is responsibile for delivering the research infrastructure and managing access to these facilities over their anticipated 15 + year lifetime (https://ukgeos.ac.uk/observatories/cheshire). The knowledge generated from this infrastructure will contribute to the responsible development of new subsurface technologies, both in the UK and internationally, and will help the UK achieve its objective of net-zero carbon emissions by 2050.

The design of the Cheshire Observatory encompasses ca. 20 instrumented boreholes drilled to a depth of ~ 100 m bgl. It includes over 1800 state-of-the-art sensors installed to monitor subsurface change in response to energy storage, including variation in groundwater flow, chemistry, temperature and microbiology. The array design also provides fibre optic Distribuited Temperature and Acoustic Sensing (DTS and iDAS) monitoring of ground temperature and motion, and resistance tomography imaging of subsurface processes. Energy would be introduced into the subsurface through heated and cooled closed loop borehole heat exchangers, with groundwater flow control via a dipole abstraction/reinjection system. The planned infrastructure will provide subsurface data that can be streamed online in real-time, through https://www.ukgeos.ac.uk/, allowing access to monitoring datasets, including those on ground motion.

Measuring ground deformation over geoenergy sites with standard geodetic instruments (e.g., GNSS) is an expensive and time-consuming task. Over the last 20 years, Interferometric Synthetic Aperture Radar (InSAR) technique has been widely used for mapping ground deformation from space due to natural hazards^1^ and to anthropogenically induced changes in (water, gas or oil) pore pressure^2–5^. Nowadays InSAR provides high spatial resolution (a few meters) and almost weekly measurements of surface deformation with millimetric accuracy. InSAR-based monitoring is less expensive, more frequent and less invasive than standard geodetic monitoring techniques and can provide appropriate monitoring at site scale^6^. Although InSAR application over geoenergy sites is well documented^7–9^, as of today no work has studied ground conditions ahead of the construction and exploitation of a site. This work documents baseline monitoring undertaken by BGS to characterise ground motion in the area of the Cheshire Observatory ahead of facility contruction. The aim of this work is to understand the association between ground motion and the geological setting of the area and to document the ongoing deformation, which can be used as baseline data against future ground motion conditions. The investigations are independent of any environmental monitoring carried out by industry or the regulators in this area, and the results made freely available. Regular monitoring of areas prone to ground motion is invaluable for locating their precursory signals on the surface, alerting stakeholders to potential disasters, mitigating their negative evolution (e.g., induced seismicity) and supporting the decision-making processes on the construction and operation of infrastructures and industrial facilities.

We focus the InSAR analysis on three periods between 1995 and 2017 and resolved ground deformation at high temporal resolution which provides a synoptic view of any precursory deformation and relationship with the existing geological conditions.

## The Cheshire Geoenergy Observatory

**Figure 1:**
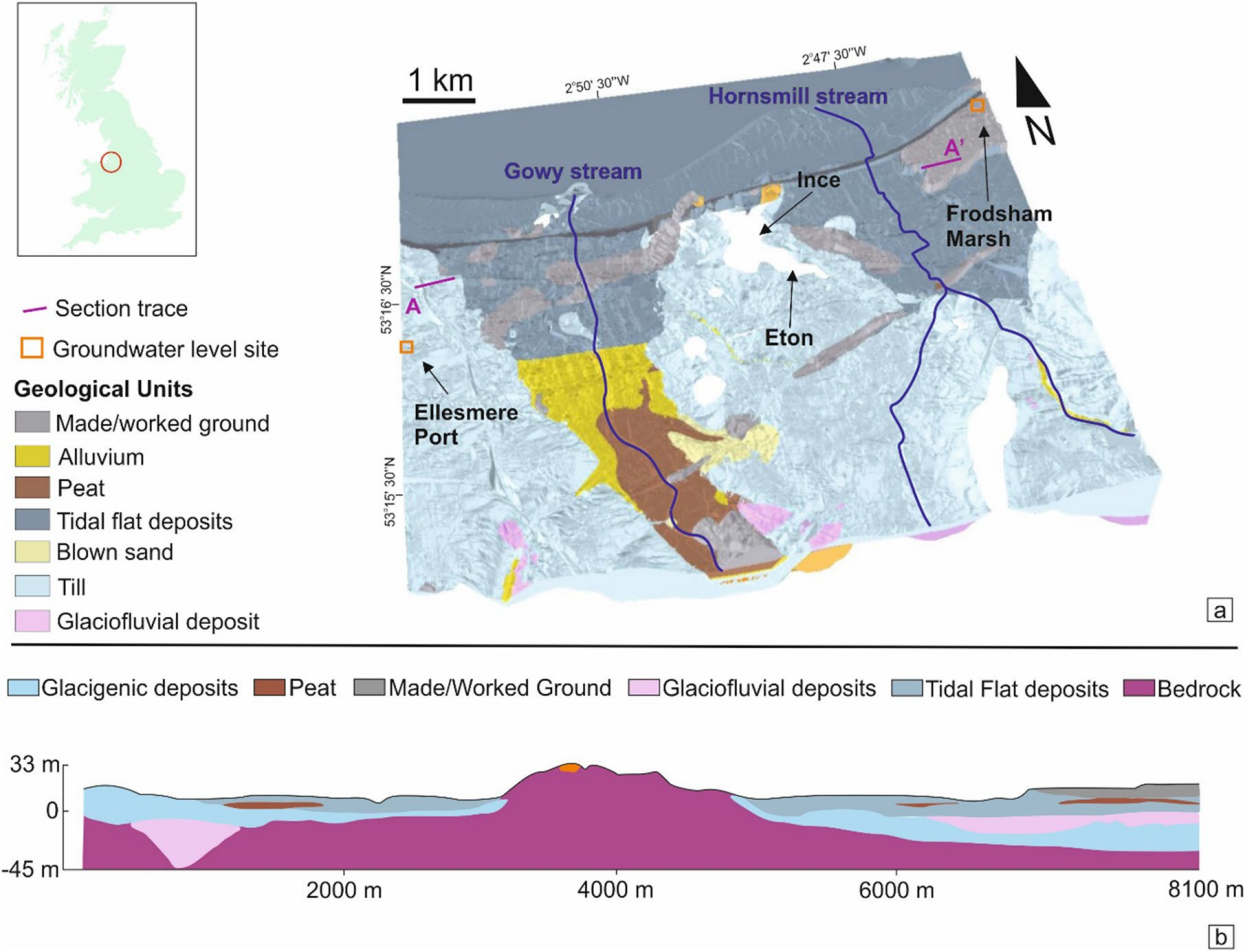
Location and geological setting of the area under consideration for the location of the Cheshire Observatory. (**a**) Location of the study area within the Great Britain and 3D superficial model of the area of interest^10^ with all geological units shown and location of the groundwater level site measurements from the Environment Agency. Vertical exaggeration 20:1. (**b**) WSW-ENE geological cross section for superficial deposits derived from the 3D geological model; vertical exaggeration 10:1. White areas indicate where these units are absent. Includes Ordnance Survey data, Crown copyright and database rights [2016] Ordnance Survey [100021290 EUL] and British Geological Survey materials, UKRI 2021.

The target area for the development of the Cheshire Geoenergy Observatory is known as the Ince Marshes, located between the Manchester Shipping Canal in the north and the M56 motorway in the south (Fig. 1), which is part of the estuarine marshes and tidal flats on the southern bank of the Mersey estuary. Extensive geological data is available for this area, including deep boreholes, 3D seismic survey data and extensive 2D seismic from the oil and coal industries (https://ukogl.org.uk/). Additionally, the area hosts a cluster of low-carbon of science and technology industries participating in the HyNet project (https://hynet.co.uk).

The upper surface of the bedrock in the target area was deeply incised during the Quaternary, with thick accumulations of superficial deposits infilling a series of broadly north-trending buried channels. The superficial deposits of the area are summarised in a 3D model^10^ and based on the Single Onshore Borehole Index (SOBI) database (Fig. 1a). The model shows that the youngest Quaternary sediments in the study area are peat, wind-blown sand, tidal flat deposits and alluvium. The tidal flat deposits and alluvium typically comprise unconsolidated sand, silt and clay and may include lenses of peat or organic-rich muds (Fig. 1a). A complex sequence of glacial deposits associated with the Late Devensian glaciation occurs at surface through much of the study area and underlies the tidal flat deposits/alluvium. These glacial deposits principally comprise till (boulder clay), consisting of gravelly clay with lenses of glacio-fluvial sand and gravel and glaciolacustrine laminated clay and silt^11^. The thickness of the superficial deposits ranges up to a maximum thickness of over 60 m, but they are completely absent beneath parts of Ince and Elton (Fig. 1b). The Quaternary succession unconformably overlies faulted Permo-Triassic sandstone bedrock, which varies in thickness from approximately 250 m to over 1000 m. The bedrock comprises the Triassic Sherwood Sandstone Group, where Environment Agency (EA) groundwater measurements are available for this area. The Sherwood Sandstone Group is underlain in some locations by the Permian Collyhurst Sandstone Formation and/or the Manchester Marls Formation. Below the Permo-Triassic succession are older Carboniferous strata, with deep boreholes proving sedimentary rocks of the Warwickshire Group, Coal Measures Group, Millstone Grit Group and Craven Group at depth^11^. The Quaternary succession is concentrated within two ancient NNW–SSE valleys filled with thicknesses of ~ 40/50 m of glacigenic and glaciofluvial deposits topped by tidal flats deposits made of sand deposits and peat levels. The buried channels broadly underlie modern drainage systems associated with Gowy and Hornsmill streams (Fig. 1b).

**Figure 2:**
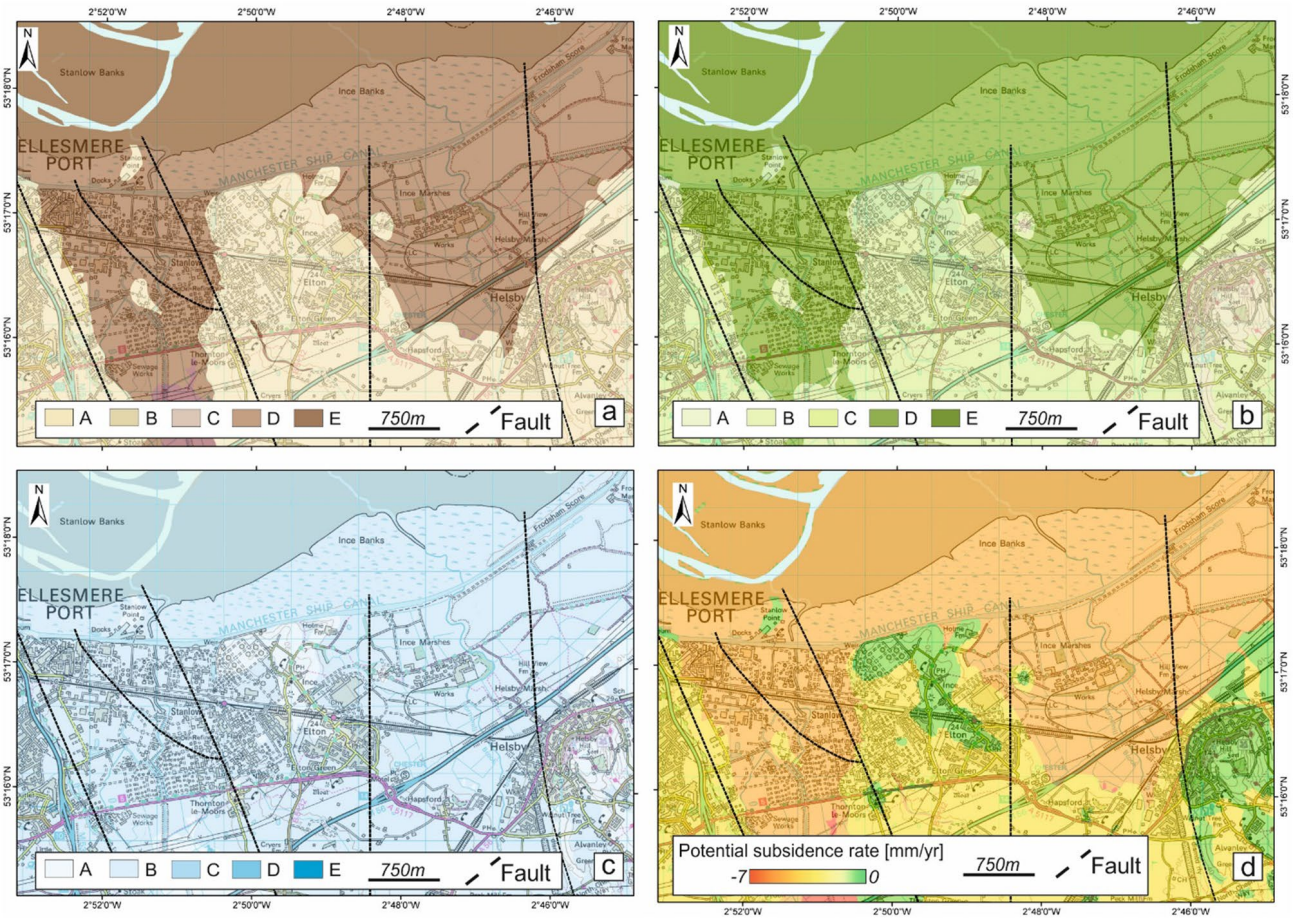
Susceptibility of the Cheshire Observatory to ground motion due to geological conditions. (**a**) GeoSure susceptibility map for compressible ground, (**b**) running sand deposits and (**c**) shrink-swell terrains. (**d**) Potential subsidence rates in the target area for the Cheshire Observatory^13^ due to the presence of compressible, soluble and shrink-swell terrains. Contains Ordnance Survey data, Crown copyright and database right 2018, license number 100021290 EUL. Contains British Geological Survey materials, UKRI 2021.

BGS-produced GeoSure susceptibility maps related to potential natural ground movement in Great Britain^12^ reveal that the area is vulnerable to instability. The GeoSure datasets are polygons representing different levels of susceptibility (from A = very low to E = very high) due to geological conditions. BGS GeoSure shows that the area is prone to movement mainly due to compressible ground (Fig. 2a) and running sand (Fig. 2b) rather than shrink and swell (Fig. 2c). The highest GeoSure ratings for compressible ground and running sand match two north-trending buried valleys following the Gowy and Hornsmill streams where peat and tidal flat deposits occur, with bedrock outcrops exhibiting low to very-low susceptibility. Running sand, differently from compressible ground, represent a serious hazards mainly when external (anthopogenic) factors disturb the natural conditions: e.g., excavation or leaking drains within sandy layers. While estimated subsidence rates for the buried valleys according to the Potential Subsidence product created using the methodology devised within the SubCoast project^13^ (Fig. 2d) are > 5 mm/year, the area does not have a dense GNSS network or frequent surveys (aerial, spaceborne, field). In such a poorly monitored setting, InSAR can provide valuable insights into ground motion trends and relationships with natural processes or anthropogenic activities.

## Results and discussion

The results from the Intermittent Small Baseline and Subset (ISBAS) analysis comprises 144,702 ERS, 34,787 ENVISAT and 153,569 Sentinel-1 pixels with all the measurements relative to a reference point placed in Liverpool near the ‘LIVE’ GNSS station and outside the studied area (see InSAR processing section for more details). LIVE belongs to the British Isles continuous GNSS Facility (BIGF) and confirms the stability of the reference point because the GNSS between 1999 and 2011 has had an average rate of motion of 0.1 ± 0.3 mm/year along the vertical direction. Results have also been validated through the DARE GNSS station where the GNSS average rate of motion was − 0.2 ± 0.2 mm/year along the vertical direction between 2000 and 2016. A threshold of 2 times the standard deviation (σ) of the distribution of the LOS velocities has been chosen to discriminate stable from unstable pixels^14^. These thresholds amount to ± 1.6 mm/year for ERS, 2.6 mm/year for ENVISAT and 1.4 mm/year for Sentinel-1. The classification of the InSAR time series has included unstable pixels only. Despite the InSAR analysis including observations only from one geometry, the geological condition of the area and its low-lying topography support the assumption that movements observed in this part of Cheshire are mainly vertical.

### ERS results

**Figure 3:**
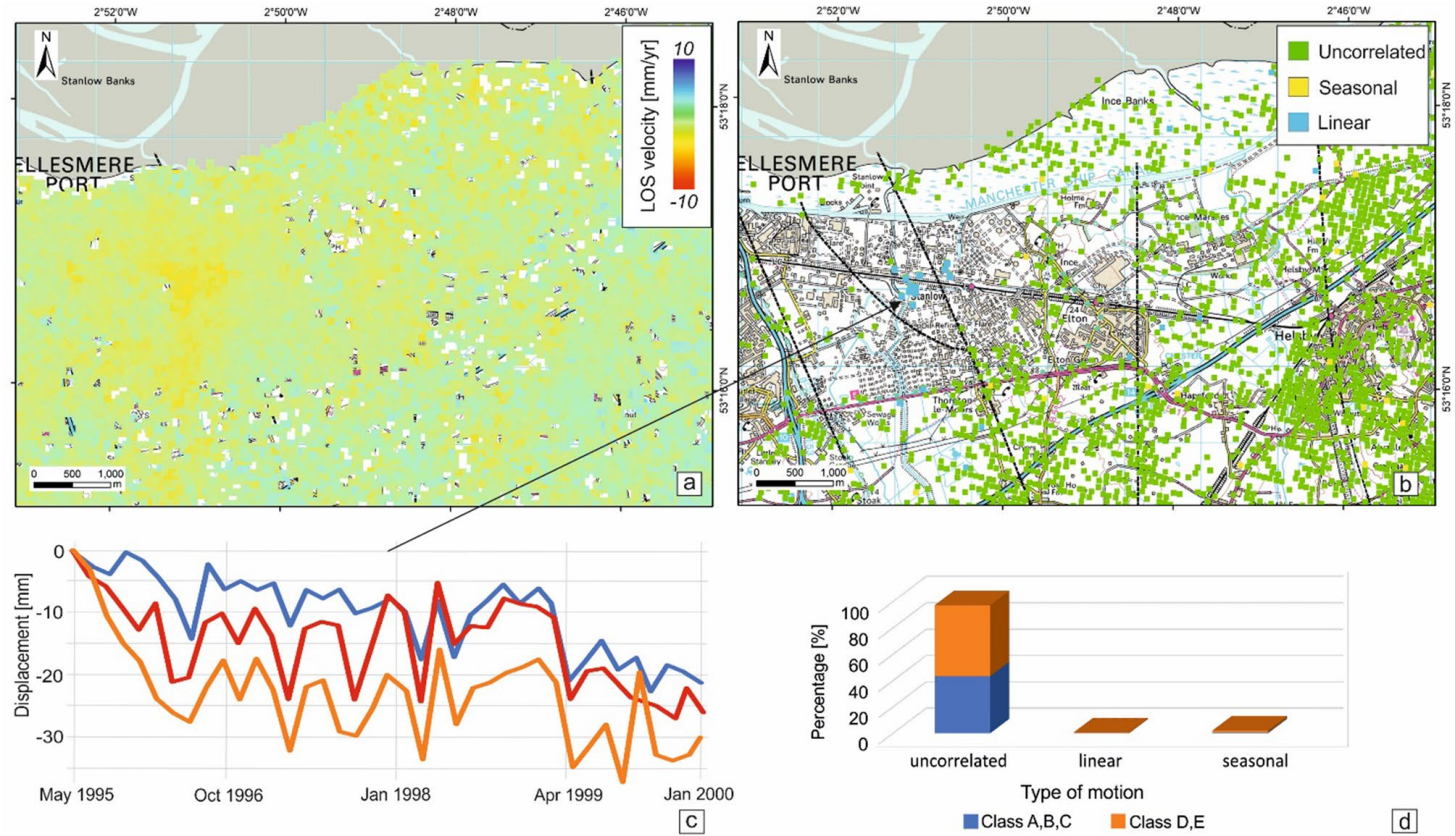
Ground stability condition for the Cheshire Observatory during 1995–2000. (**a**) ISBAS results from ERS data, (**b**) classification of the unstable InSAR time series, (**c**) time series for four ISBAS points within the study area and (**d**) percentage of type of motion according to the GeoSure susceptibility classes in the compressible ground and running sand susceptibility layers. Contains Ordnance Survey data, Crown copyright and database right 2018, license number 100021290 EUL. Contains British Geological Survey materials, UKRI 2021. Contains Terra Motion Limited 2017 data.

The ISBAS results from ERS-1/2 data indicate that the majority of the area was stable for the time period 1995–2000 (Fig. 3). A minor subsidence bowl is present in the west of the study area (south of Ellesmere Port) where maximum velocities of up to 6 mm/year occur (Fig. 3a). The subsiding area extending for ~ 1.5 km^2^, is located on the western edge of the target area and coincides with the high-susceptibility to compressible ground and running sands identified in GeoSure (see Fig. 2). When the motions are classified, it is revealed that most of the (unstable) time series are uncorrelated (Fig. 3b), a phenomenon that can be connected to the temporal decorellation between the images acquisition (43 days on average). Targets at the centre of the subsiding bowls show a linear motion (Fig. 3c) that, however, represents a minor percentage (~ 2%) of the type of deformation patterns (Fig. 3d).

### ENVISAT results

**Figure 4:**
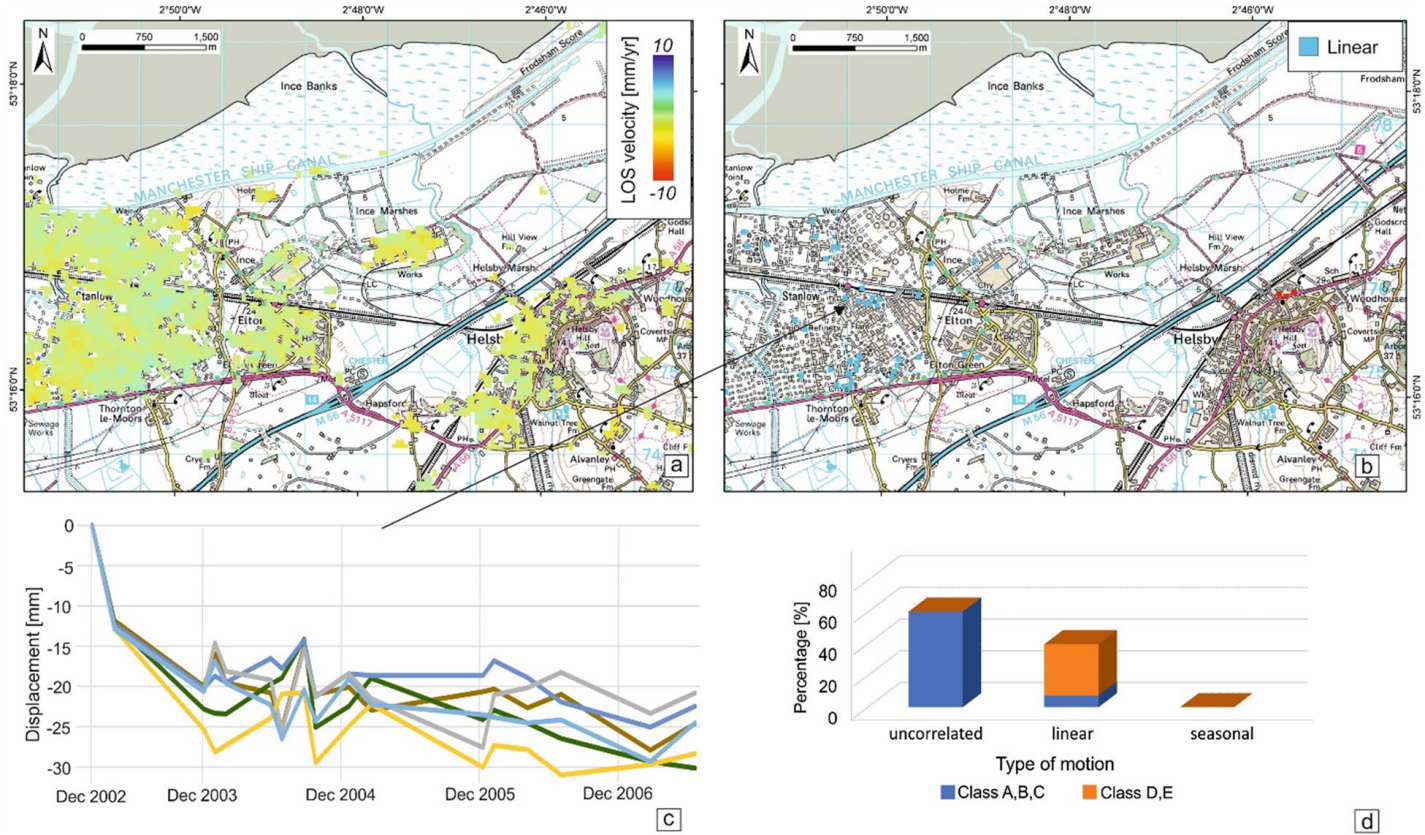
Ground stability condition for the Cheshire Observatory during 2002–2007. (**a**) ISBAS results from ENVISAT data, (**b**) classification of the unstable InSAR time series, (**c**) time series for five ISBAS points within the study area and (**d**) percentage of type of motion according to the GeoSure susceptibility classes in the compressible ground and running sand susceptibility layers. Contains Ordnance Survey data, Crown copyright and database right 2018, license number 100021290 EUL. Contains British Geological Survey materials, UKRI 2021. Contains Terra Motion Limited 2017 data.

The ISBAS results from ENVISAT data (2002–2007) have a lower density of InSAR measurements, from 138 ISBAS points/km^2^ during the ERS period to 30 ISBAS points/km^2^. The lower density can be ascribed to the low temporal frequency of SAR images aquired during this period (every 3 months on average). The lower coverage reduces the reliability of the results by increasing the standard deviation of the LOS velocity population but still provides enough coverage over the target area for our baseline study. The area under consideration for the Cheshire Observatory is stable overall (Fig. 4a) with the unstable InSAR time series displaying a linear pattern of motion (Fig. 4b). The linear motion of ISBAS points on the western sector of the observatory shows a subsidence range of − 5 to − 7 mm/year (Fig. 4c). The subsidence looks quite rapid, ≥ 10 mm, during December 2002 and December 2003, which we believe is real motion also affected by errors induced by the high temporal baselines (up to 8 months) between the available image acquisitions. The subsidence bowl is spatially less pronounced than in the ERS period given the lower average velocities but the linear trend now characterizes 86% of the unstable pixels while it was < 1% during the ERS period (Fig. 4d).

### Sentinel-1 results

**Figure 5:**
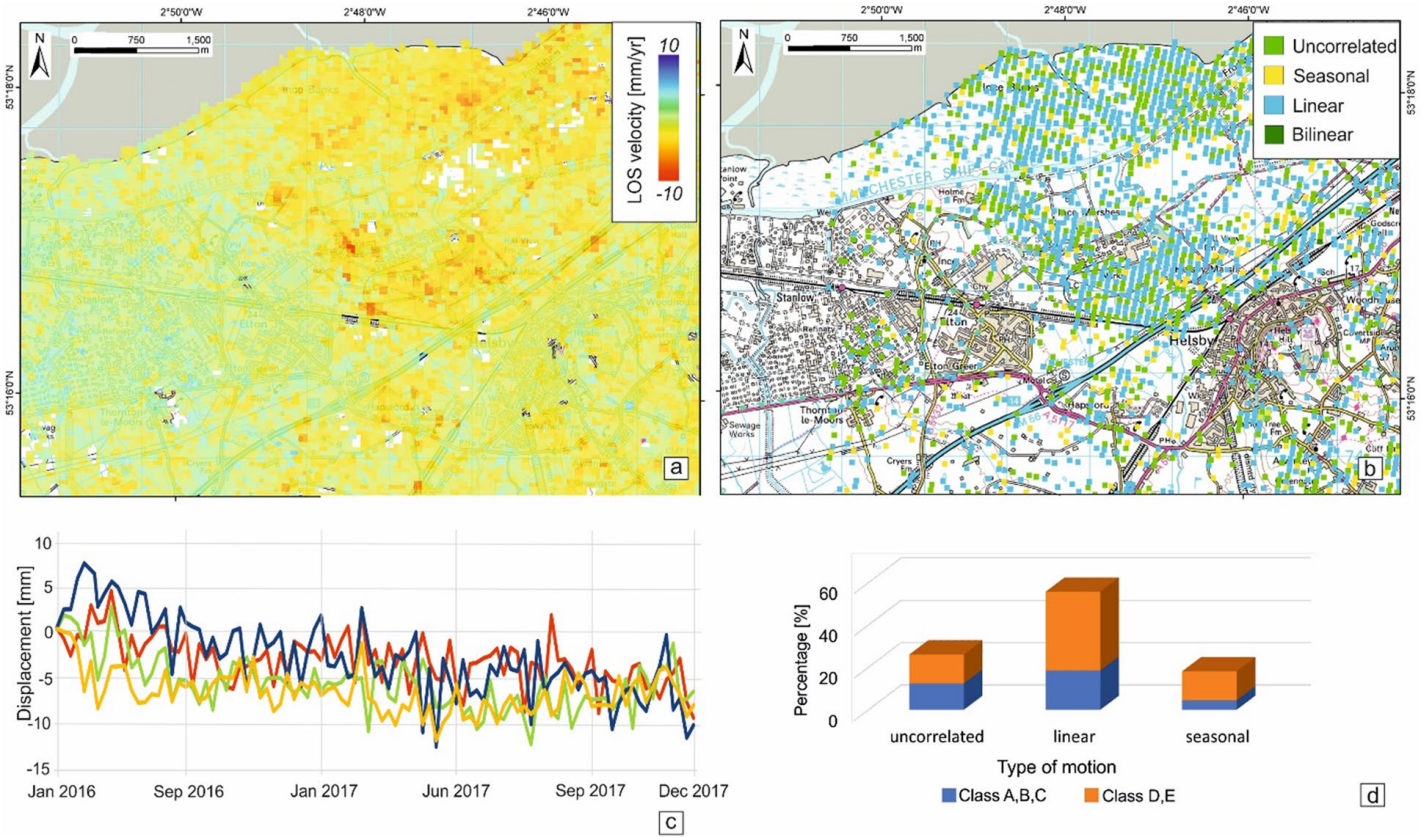
Ground stability condition for the Cheshire Observatory during 2016–2017. (**a**) ISBAS results from Sentinel-1 data, (**b**) classification of the unstable InSAR time series, (**c**) time series for four ISBAS points within the study area and (**d**) percentage of type of motion according to the GeoSure susceptibility classes in the compressible ground and running sand susceptibility layers. Contains Ordnance Survey data, Crown copyright and database right 2018, license number 100021290 EUL. Contains British Geological Survey materials, UKRI 2021. Contains Terra Motion Limited 2017 data.

The Sentinel-1 processing provides results with a spatial density of 142 ISBAS points/km^2^. As with the previous datasets, the processed area remains stable at large scale. However, the InSAR data detected subsidence associated with the N–S oriented paleochannels that run through the study area (Fig. 5a). The high frequency of the Sentinel-1 acquisitions (up to 6 days) allows the detection of patterns of motion at greater detail than possible with ERS and ENVISAT. A major proportion of the ISBAS points in the study area show a linear subsidence type of motion (Fig. 5b) with velocity as much as − 10 mm/year (Fig. 5c). On the other hand, targets with seasonal patterns (~ 17% of the total) show different wavelengths and are scattered, therefore we can exclude any common source of ground motion. Spatially, the subsidence pattern characterises the areas that GeoSure has identified as most vulnerable to ground motion due to the existing geological predisposing factors. The higher revisiting time of Sentinel-1 provides a much clearer spatial separation, compared to ERS and ENVISAT results, of the different type of motions with the subsidence pattern with the lowest velocities now concentrated in the middle of the study area where the majority of pixels (~ 56%) have a linear pattern of motion (Fig. 5d).

### Probable source of the ground deformation

**Figure 6:**
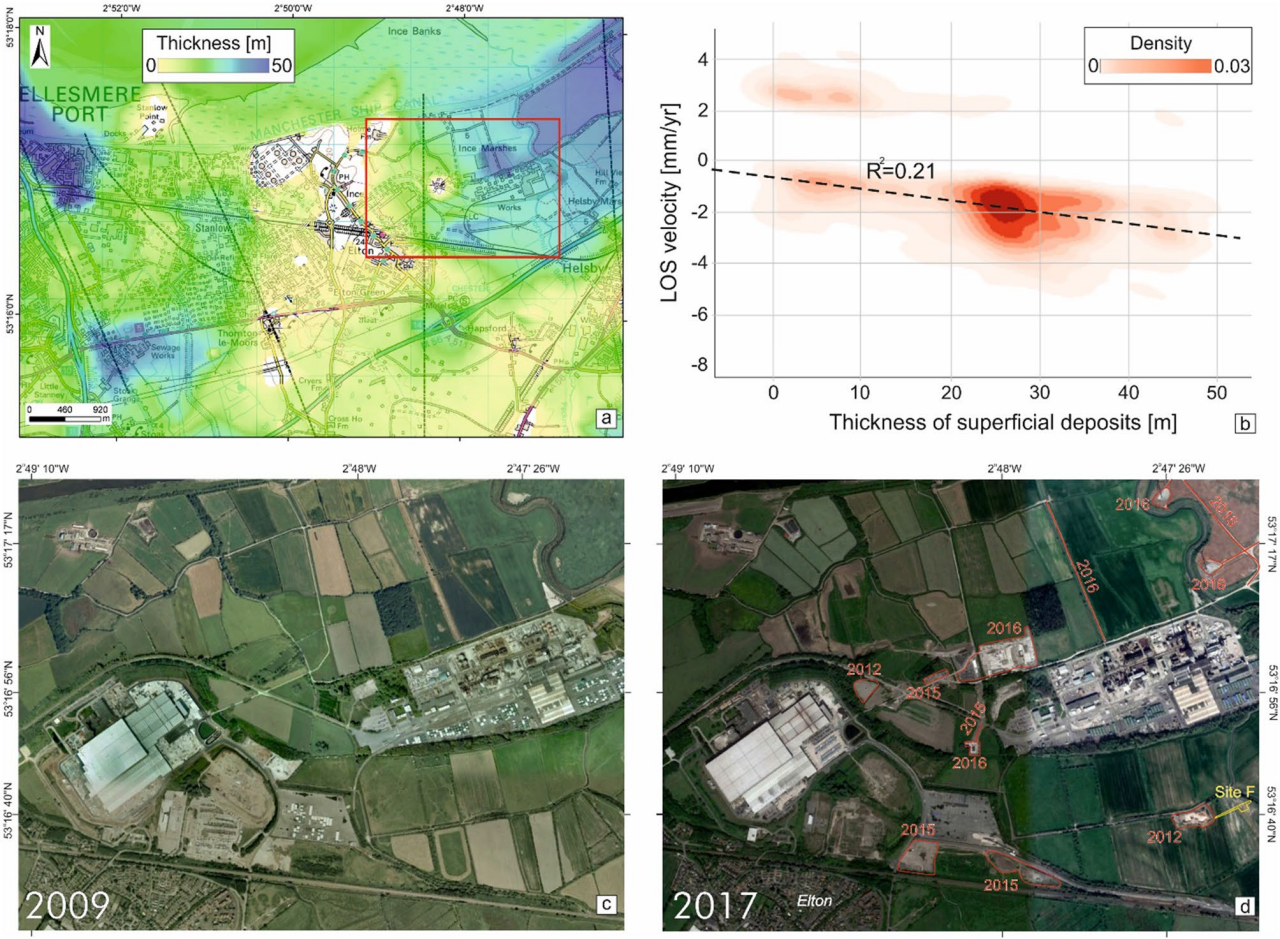
Correlation of ground motion with superficial deposit thickness and contruction activities. (**a**) thickness map of the superficial deposits. (**b**) kernel density estimate plot of the LOS velocity of the unstable targets with a linear pattern of motion identified in the ERS, ENVISAT and Sentinel-1 InSAR results. Major land cover changes observed between (**c**) December 2009 and (**d**) April 2017 within the studied area (red box in **a**). Maps data: Google, 2021 Maxar Technologies, 2021 Infoterra Ltd & Bluesky Image and 2021 Getmapping plc.

In this study InSAR baseline monitoring has been undertaken for the area under consideration for the development of the Cheshire UK Geoenergy Observatory. This has been done to determine pre-operational ground stability between 1995 and 2017. A main area of motion has been identified in the north-eastern part of Ince Marshes commencing in 2003 but accelerating from 2016. The consistency of the observed motion patterns between the three independent displacement maps provides verification that this area is prone to the same form of deformation over a period of 23 years. We have therefore analysed the ERS, ENVISAT and Sentinel-1 linear patterns of motion in relation to the modelled thickness of the superficial deposits^10^ (see Fig. 1), recent construction activities, and hydrogeological conditions (Fig. 6a). We have compared the thickness values vs the LOS velocities of the linear patterns of motion of the unstable targets with the kernel density estimate (Fig. 6b) showing a positive correlation (R^2^ = 0.21). In addition to geological factors, twelve new buildings and associated infrastructure have been constructed in the eastern part of the study area since 2009 (Figs. 6c,d). This period corresponds to soon after the ENVISAT observations and includes most of the Sentinel-1 observations. The cumulative area of the new construction amounts to ~ 0.11 km^2^ (< 1% of the study area) and mainly relate to facilities associated with innovative technologies in energy generation and resource management.

**Figure 7:**
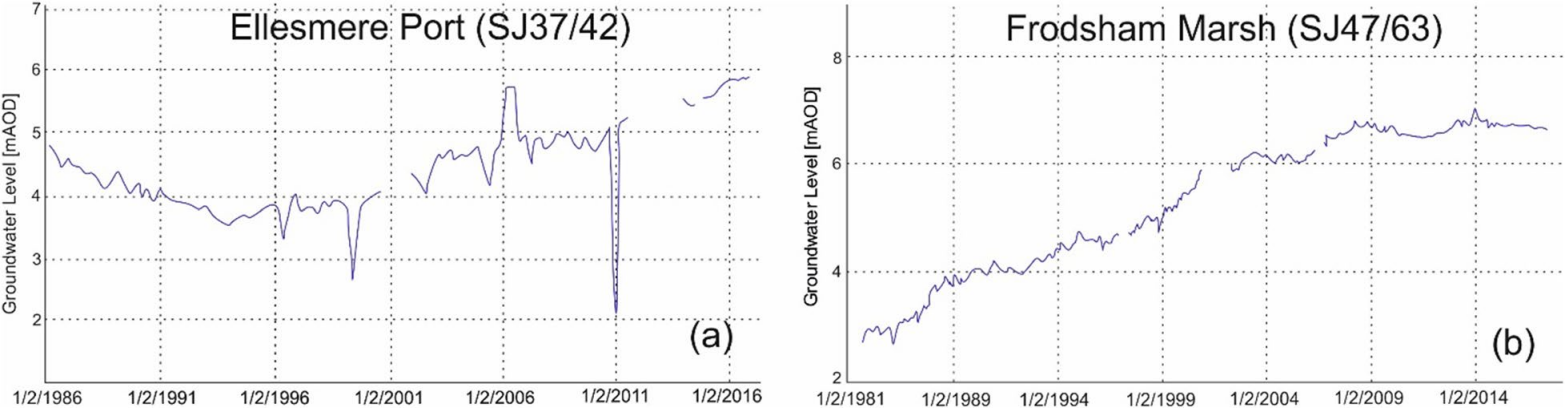
Groundwater levels in the Cheshire Observatory area. Groundwater level for (**a**) Ellesmere Port and (**b**) Frodsham Marsh. For their location see Fig. 1. Contains Environment Agency information.

Time series of groundwater levels from the closest EA borehole sites have also been analysed (Fig. 7). We believe that the ground motion at surface cannot be related to groundwater variations. Indeed, these changes show a gradual uplift over the past 25 years, have a small magnitude (in the order of few meters) and occur only in the bedrock.

Collection, interpretation, and processing of geo-thematic, groundwater and landcover data were fundamental to determine the potential cause(s) of this motion. The wealth of ancillary datasets available for the target area has facilitated our geological interpretation and we acknowledge that this holistic approach might be challenging to perform in a data-poor environment.

The subsidence at the Ince Marshes shows a strong linearity in time and spatially concentrated in an area already known for its highly vulnerability to compressibility given the underlying superficial deposits, especially where tidal flat deposits with peat intervals and loosely-packed sandy layers are present in the near-surface. In this regard, the buried channels in the bedrock have inevitably played a major role representing a geomorphological pre-condition for a thicker deposition of poorly consolidated sediments whose consolidation is driving the subsidence in the area. More generally, the wide area analysis (not shown in this paper) confirms that subsidence is also affecting tidal flat and peat deposits along the Mersey Estuary. Faults do not appear to have a role in the subsidence because they do not cut through the superficial deposits^10^ and the ones in the bedrock are not spatially correlated with the ground motion of the area. We exclude any displacement connected to changes in soil moisture given the low susceptibility of the area to shrink-swell and the lack of predominant clay materials within the superficial deposits which have thicknesses of ~ 3 m within the subsiding area. We can also exclude mine collapse phenomenona given the absence of any present and past coalfield here and in the surrounding area even if such scenario is one of the major causes of ground deformation in Great Britain^15, 16^. On the other hand, natural consolidation processes^17^ which might be quite rapid as observed during the start of the ENVISAT period, and the loading of the ground by new buildings and infrastructure is a recognised mechanism for subsidence that has also been studied with InSAR^18^. It is probable that, on top of consolidation phenomena, the recent construction contributed to the observed subsidence or, at least, to the acceleration in the InSAR time series observed for 2016–2017 considering that the thickness of the superficial deposits has not changed in recent times. Because information on buildings’ foundation type and depth were not available, we cannot exclude or assess the compaction effect of the new infrastructure to the observed subsidence. However, we infer that modern buildings have been designed and constructed with appropriate foundations for such well-known compressible ground conditions and it is therefore unlikely that direct loading by the new building has resulted in motions but the loading of the ground during the construction stage and disruption of the water table may account for the construction-related motion^19^. The availability of data on the foundations of these buildings can provide further evidence to support our theory. Despite InSAR observations spanning three decades, the long revisit time of ERS and ENVISAT represents a limitation for a clear understanding of the pattern of motion of the targets and, then, to the interpretation of the results especially at local scale. On the other hand, further years of Sentinel-1 observations will provide information to assess the (medium and long term) influence of recent construction on the observed subsidence and disentangle, for example, the presence of any primary/secondary consolidation effects. In order to take advantage of future Sentinel-1 acquisitions, BGS has designed an array of artificial reflectors to be installed over rural areas to increase InSAR measurement points. This will allow the analysis of ground stability conditions at even higher accuracy and precision than previously over a geoenergy site.

## Conclusions

This study has shown that analysing InSAR data in combination with geodata can yeld important insights into controls on ground motion and help differentiate natural ground motion from that related to anthropogenic activities. We envisage that the methodology can be used as a best-practice protocol for baseline monitoring and can provide the detailed baseline information needed to reassure stakeholders, including the public and regulatory bodies^20, 21^. InSAR data from the Cheshire Observatory site will be published online on the UKGEOS website (https://www.ukgeos.ac.uk/) when available, to provide long term transparent monitoring information.

## Methods and datasets

**Figure 8:**
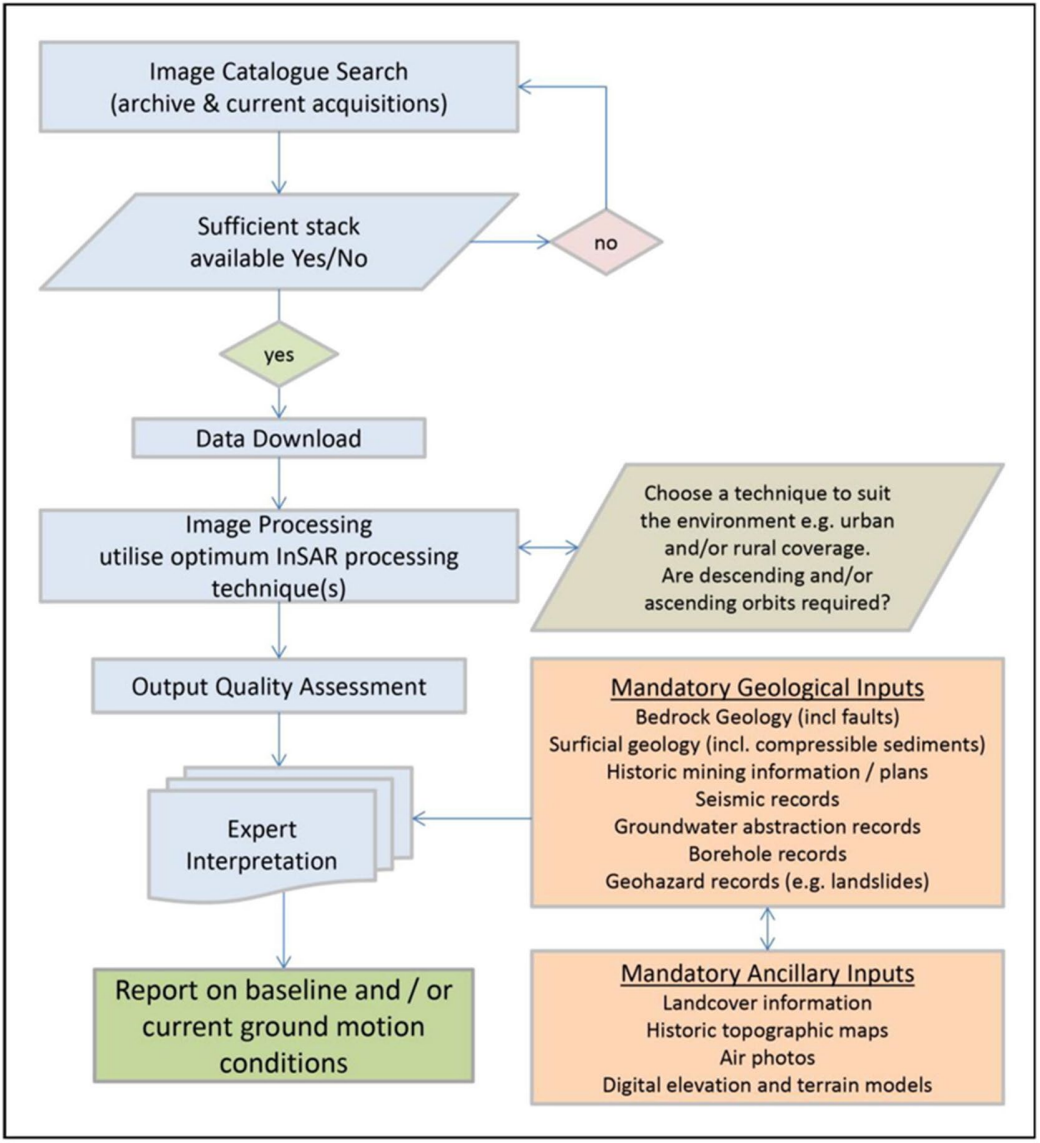
Flowchart of the approach and data utilised for the ground motion InSAR monitoring^6^.

The approach followed for acquiring, processing and interpreting the InSAR data is based on an existing workflow^6, 20^ shown in Fig. 8. The approach integrates, where possible, InSAR data with other geological, geophysical and hydrogeological datasets to support the interpretation not just at a local scale but also over a wider area which might be directly or indirectly affected by underground operations as well.

### InSAR processing

InSAR data is processed by analyzing the phase difference across a series of radar images aquired over the same location at different times, and using this to retrieve millimetric changes in ground motion^22^. The motion of each pixel is relative in space to the satellite Line of Sight (LOS) direction and relative in time to a reference point deemed to be stable over time. The reference point (53° 24′ 13″ N and 2° 58′ 24″ W) in this case has been located near the LIVE GNSS station (53° 26′ 59″ N and 3° 1′ 5″ W). Validation of the LOS displacements has used the DARE station located at 53° 20′ 41″ N and 2° 38′ 26″ W. For the analysis we retrieved historical satellite acquisitions back to the 1990s and consisting into three descending SAR stacks covering an area of ~ 1000 km^2^ over a time span of 23 years:

- 37 ERS-1 and ERS-2 images (from 8th May 1995 to 18th January 2000, respectively).
- 17 ENVISAT images (from 3rd December 2002 to 5th June 2007).
- 95 Sentinel-1A and Sentinel-1B images (from 8th January 2016 to 28th December 2017).

The investigation focused on a small portion of the entire SAR scene, whose footprint is 10,000 km^2^ for ERS and ENVISAT and ~ 45,000 km^2^ for Sentinel-1, and includes the area under consideration for the development of the Cheshire Observatory. Satellite radar data were downloaded from the Copernicus Open Access Hub (https://scihub.copernicus.eu/dhus/#/home) of the European Space Agency and processed by Terra Motion Limited using a technique known as ISBAS^23^. This method was chosen due to its ability to provide a higher density of measurements over rural areas^24^. Retrieving ground motion information for non-urban and semi-vegetated areas using standard InSAR approaches with C-band (~ 5.6 cm wavelength) satellite radar imagery is challenging due to sources of noise like temporal decorrelation^25^. The ISBAS algorithm^26^ is very similar in structure to the SBAS algorithm^27^ where, from a stack of multiple SAR observations, a large number of differential interferograms are generated, satisfying a spatial threshold that primarily relates to a low orbital baseline. The baselines chosen have been 1000 m for ERS and ENVISAT and 500 m for Sentinel-1. In standard SBAS, the phase of pixels that are of a consistently high coherence (γ) throughout the stack are then examined through regression analysis to estimate linear velocity and a height correction for the digital elevation model (DEM) used for the two-pass interferometry.

However, ISBAS modifies the approach for selecting image pixels to process by relaxing the threshold that the pixel must display consistently high coherence over all interferograms. The chosen threshold, of ≥ 0.25, accounts for the intermittent nature of non-urban targets and allows the extension of the coverage of motion results across the full range of land cover types, even those typically unfavourable for InSAR^28^. To increase the phase signal quality and reduce radar speckle the scenes were multi-looked. The DTM used for the height correction and geolocation is the 90 m product available from the Shuttle Radar Topography Mission (SRTM^29^). Phase unwrapping of the interferograms was conducted using the Statistical-cost Network-flow Algorithm for Phase Unwrapping (SNAPHU) programme^30^ to individually unwrap coherent pixels within each interferogram. Following the unwrapping process, linear velocity and displacement time-series for the coherent pixels were computed using a regression analysis.

Both average velocities and time-series of deformation are calculated along the satellite LOS for each pixel with a ground distance of ~ 100 m for ERS and ENVISAT and of ~ 88 m for Sentinel-1. The LOS incidence angle from the vertical at the centre of the SAR scene is 23.3° for ERS and ENVISAT and is 37.5° for Sentinel-1. Such values make the LOS more sensitive to the vertical motion rather than the horizontal one. The validation of the InSAR results usually consider ground truth data such as other independent geodetic reference measurements. In this case we used the stations of the NERC BIGF archive. InSAR analysis is important to temporally and spatially constrain ground instability and to determine the potential or most likely cause(s) of the motion. However, a major challenge for using InSAR for the measurement of deformation signals is the identification and separation of the different sources of motion, especially when ten of thousands of different pixels have to be analysed. We have therefore used a tool^31^ to automatically classify InSAR time series into distinctive patterns. The analysis is done in Octave and includes a sequence of statistical tests (e.g., ANOVA and Bayesian Information Criterion) that discriminate different styles of ground deformation and classify the InSAR time series in one of predefined target trends such as uncorrelated (displacement fluctuates erratically over time), linear (linear and constant velocity) and non-linear (changes of the velocity over time and style of deformation that can be quadratic, bilinear, discontinuous with constant velocity and discontinuous with variable velocity). The identification of these patterns supports the interpretation of the InSAR data that can be more easily linked to the different sources of deformation when ancillary datasets are analysed^32^.

### Ancillary data analysis

For the studied area, 203 SOBI boreholes have been used to derive and constraint the total thickness of the superficial deposits^10^. The superficial deposits thickness map was calculated by subtracting the combined bases of all modelled Quaternary and anthropogenic units from the Digital Terrain Model and clipping the resultant grid to the extent of modelled Quaternary and anthropogenic units. The 3D model is based on information provided in the BGS Geology dataset, a GIS layer available at different scales of geological maps of polygons of the bedrock geology, superficial deposits, mass movement and artificial ground labelled or attributed according to their lithostratographical, chronostratographical or lithodemic nomenclature or their composition. The BGS GeoSure national datasets^12^ provide geological information about potential ground movement or subsidence that can help planning decisions and it is based on the BGS Geology dataset. The compressible ground layer covers the ground motion and consider the presence of weak materials like peat or clays while the running sand layer considers rocks and soils containing loosely packed sandy layers that can become fluidised by water flowing through them. The SubCoast Potential Subsidence product shows the potential for European coastal zone areas to undergo subsidence due to the natural processes of compaction, dissolution and shrinkage of geological deposits^13^ and uses layer composition and thicknesses from the BGS Geology data. This methodology was used by BGS to determine which geological deposits might undergo these processes and calibrate it against InSAR velocities at specific locations and then use the correlation to map potential subsidence rates across the coastline of Great Britain. Correlations between InSAR average velocities and the thickness map has been done by extracting the kernel density estimate between these two variables.

The Coal Authority collection contains coal and other mineral abandonment plans, covering both surface and deep mining operations, which depict areas of coal and other mineral extraction and the point of entry into these workings. These plans being referenced by graticule squares based on the old County Series Plans. Areas of abandoned coal mining represent one of main source of motion in Great Britain^33^ with ground uplift is the result of previously pumped mine water allowed back into the workings^34, 35^.

The EA maintains a network of 3 groundwater level observation boreholes within the investigated area with historical data dated back to 1969 and hourly time series data for groundwater levels available since December 2016. Only Ellesmere Port and Frodsham Marsh site have observation available for the InSAR time span.

## Data Availability

ERS data corresponds to track 409 and frame 2529, the data was downloaded from the EOLI-SA Catalogue of the European Space Agency (ESA). ENVISAT data corresponds to track 409 and frame 2529, the data was downloaded from the ESA EOLI-SA Catalogue. The Sentinel-1 datasets are freely available and can be obtained by searching and downloading the Interferometric Wide (IW) swath mode products for orbit track numbers 52 through the Copernicus Open Access Hub (https://scihub.copernicus.eu/dhus/#/home). The InSAR datasets and classified time series may be made available on request by email addressed to the corresponding author (A.N.) along with the code used to classify the InSAR time series. Geological data of bedrock and superficial deposits belongs to the BGS Geology 50 K data at 1:50,000 scale^36^ and the recently developed 3D Quaternary geology model for Cheshire^10^. The product can be requested at https://www.bgs.ac.uk/datasets/bgs-geology-50k-digmapgb/. GeoSure data on the underlying geological conditions of the Cheshire Observatory can be requested at https://www.bgs.ac.uk/datasets/geosure/. SOBI is an index of over 1 million boreholes, shafts and wells and references collections of digital and analogue records from all forms of drilling and site investigation work held by the BGS and freely available at https://www.bgs.ac.uk/datasets/boreholes-index/. SubCoast data may be made available on request by email addressed to the corresponding author (A.N.). The abandoned mines catalogue from the CA database available at http://mapapps2.bgs.ac.uk/coalauthority/home.html. Finally, groundwater level data have been made available upon request to the EA.

## References

[1] Biggs J, Wright TJ (2020). How satellite InSAR has grown from opportunistic science to routine monitoring over the last decade. Nat. Commun..

[2] Vasco DW (2010). Satellite-based measurements of surface deformation reveal fluid flow associated with the geological storage of carbon dioxide. Geophys. Res. Lett..

[3] Chen RZ (2011). Poroelastic model for induced stresses and deformations in hydrocarbon and geothermal reservoir. J. Petrol. Sci. Eng..

[4] Shirzaei M, Ellsworth WL, Tiampo KF, González PJ, Manga M (2016). Surface uplift and time-dependent seismic hazard due to fluid injection in eastern Texas. Science.

[5] Kim JW, Lu Z (2018). Association between localized geohazards in West Texas and human activities, recognized by Sentinel-1A/B satellite radar imagery. Sci. Rep..

[6] Jordan C, Bateson L, Novellino A (2019). Environmental baseline monitoring for shale-gas development: Insights for monitoring ground motion using InSAR analysis. Sci. Total Environ..

[7] Sowter A, Athab A, Novellino A, Grebby S, Gee D (2018). Supporting energy regulation by monitoring land motion on a regional and national scale: A case study of Scotland. Proc. Inst. Mech. Eng. Part A J. Power Energy.

[8] Bateson, L. & Novellino, A. Glasgow Geothermal Energy Research Field Site: Ground motion survey report. Nottingham, UK, British Geological Survey. (OR/18/054) (2019). https://www.ukgeos.ac.uk/data/publications (Accessed 10 Feb 2021).

[9] Sadeghi Z (2021). Benchmarking and inter-comparison of Sentinel-1 InSAR velocities and time series. Remote Sens. Environ..

[10] Burke, H.F. *et al.* The 3D Quaternary geology of the area around Thornton, Cheshire. British Geological Survey. (OR/16/056) (unpublished). (2016) http://nora.nerc.ac.uk/id/eprint/516445/ (Accessed 9 Dec 2020).

[11] Kingdon, A., Fellgett, M.W. & Spence, M.J. UKGEOS Cheshire Energy Research Field Site—Science Infrastructure. Nottingham, UK, British Geological Survey. (OR/19/052). (2019) http://nora.nerc.ac.uk/id/eprint/525100/1/OR19052.pdf (Accessed 10 Nov 2020).

[12] Lee, K.A. & Doce, D.D. User Guide for the British Geological Survey GeoSure dataset: Version 8. British Geological Survey, OR/17/050. (2017) http://nora.nerc.ac.uk/id/eprint/518790/ (Accessed 11 Nov 2020).

[13] Bateson, L., Evans, H. & Jordan, C. GMES-service for assessing and monitoring subsidence hazards in coastal lowland areas around Europe. SubCoast D3.5.1. British Geological Survey. (OR/11/069). (2011) http://nora.nerc.ac.uk/id/eprint/16251/ (Accessed 23 Feb 2021).

[14] Barra A (2017). A methodology to detect and update active deformation areas based on sentinel-1 SAR images. Remote Sens..

[15] Bateson L, Cigna F, Boon D, Sowter A (2015). The application of the Intermittent SBAS (ISBAS) InSAR method to the South Wales Coalfield, UK. Int. J. Appl. Earth Observ. Geoinf..

[16] Anantrasirichai N (2020). Detecting ground deformation in the built environment using sparse satellite InSAR data with a convolutional neural network. IEEE Trans Geosci. Remote Sens..

[17] Kim SW, Wdowinski S, Dixon TH, Amelung F, Kim JW, Won JS (2010). Measurements and predictions of subsidence induced by soil consolidation using persistent scatterer InSAR and a hyperbolic model. Geophys. Res. Lett..

[18] Ciampalini A, Solari L, Giannecchini R, Galanti Y, Moretti S (2019). Evaluation of subsidence induced by long-lasting buildings load using InSAR technique and geotechnical data: The case study of a Freight Terminal (Tuscany, Italy). Int. J. Appl. Earth Observ. Geoinf..

[19] Novellino, A., Terrington, R., Christodoulou, V., Smith, H. & Bateson, L. Ground motion and stratum thickness comparison in Tower Hamlets, London. Nottingham, UK, British Geological Survey. (OR/19/043). (2019) http://nora.nerc.ac.uk/id/eprint/525619/ (Accessed 15 Feb 2021).

[20] Ward, R.S. *et al.* Preliminary assessment of the environmental baseline in the Fylde, Lancashire. Nottingham, UK, British Geological Survey. (OR/18/020). (2018) http://nora.nerc.ac.uk/id/eprint/519977/ (Accessed 10 Nov 2020).

[21] Ward, R.S. *et al.* Recommendations for environmental baseline monitoring in areas of shale gas development. Nottingham, UK, British Geological Survey. (OR/18/043) (Unpublished). (2020) http://nora.nerc.ac.uk/id/eprint/528641/ (Accessed 8 Dec 2020).

[22] Rosen PA (2000). Synthetic aperture radar interferometry. Proc. IEEE.

[23] Sowter A, Bateson L, Strange P, Ambrose K, Syafiudin MF (2013). DInSAR estimation of land motion using intermittent coherence with application to the South Derbyshire and Leicestershire coalfields. Remote Sens. Lett..

[24] Grebby S (2021). Advanced analysis of satellite data reveals ground deformation precursors to the Brumadinho Tailings Dam collapse. Commun. Earth Environ..

[25] Agram PS, Simons M (2015). A noise model for InSAR time series. J. Geophys. Res. Solid Earth.

[26] Sowter A (2016). Mexico City land subsidence in 2014–2015 with Sentinel-1 IW TOPS: Results using Intermittent SBAS (ISBAS) technique. Int. J. Appl. Earth Observ. Geoinf..

[27] Berardino P, Fornaro G, Lanari R, Sansosti E (2002). A new algorithm for surface deformation monitoring based on small baseline differential SAR interferograms. IEEE Trans. Geosci. Remote Sens..

[28] Cigna F, Sowter A (2017). The relationship between intermittent coherence and precision of ISBAS InSAR ground motion velocities: ERS-1/2 case studies in the UK. Remote Sens. Environ..

[29] Farr TG, Kobrick M (2000). Shuttle Radar Topography Mission produces a wealth of data. Eos Trans. Am. Geophys. Union.

[30] Chen CW, Zebker HA (2001). Two-dimensional phase unwrapping with use of statistical models for cost functions in nonlinear optimization. JOSA A..

[31] Berti M, Corsini A, Franceschini S, Iannacone JP (2013). Automated classification of Persistent Scatterers Interferometry time series. Nat. Hazard..

[32] Bonì R (2018). A methodology to detect and characterize uplift phenomena in urban areas using Sentinel-1 data. Remote Sens..

[33] Novellino A (2017). Assessing the feasibility of a national InSAR ground deformation map of Great Britain with Sentinel-1. Geosciences.

[34] Gee D (2017). Ground motion in areas of abandoned mining: Application of the Intermittent SBAS (ISBAS) to the Northumberland and Durham Coalfield, UK. Geosciences..

[35] Gee D (2020). Modelling groundwater rebound in recently abandoned coalfields using DInSAR. Remote Sens. Environ..

[36] Armstrong, R. A., Daley, D. L., Lawley, R., Myers, A. H. & Smith, A. User Guide for the BGS Geology: 50k dataset (V8). British Geological Survey Open Report, OR/16/46. https://nora.nerc.ac.uk/id/eprint/515821/ (2016). (Accessed 10 Nov 2020).

